# Liver-derived human mesenchymal stem cells: a novel therapeutic source for liver diseases

**DOI:** 10.1186/s13287-016-0330-3

**Published:** 2016-05-12

**Authors:** Yini Wang, Xiaopeng Yu, Ermei Chen, Lanuan Li

**Affiliations:** State Key Laboratory for the Diagnosis and Treatment of Infectious Diseases, Collaborative Innovation Center for the Diagnosis and Treatment of Infectious Diseases, First Affiliated Hospital, School of Medicine, Zhejiang University, Hangzhou, 310003 China

**Keywords:** Mesenchymal stem cells, Cell therapy, Hepatic differentiation, Liver-derived mesenchymal stem cells

## Abstract

Mesenchymal stem cells (MSCs) represent an attractive cell type for research and therapy due to their ability to proliferate, differentiate, modulate immune reactions, and secrete trophic factors. MSCs exist in a multitude of tissues, including bone marrow, umbilical cord, and adipose tissues. Moreover, MSCs have recently been isolated from the liver. Compared with other MSC types, liver-derived human MSCs (LHMSCs) possess general morphologies, immune functions, and differentiation capacities. Interestingly, LHMCSs produce higher levels of pro-angiogenic, anti-inflammatory, and anti-apoptotic cytokines than those of bone marrow-derived MSCs. Thus, these cells may be a promising therapeutic source for liver diseases. This paper summarizes the biological characteristics of LHMSCs and their potential benefits and risks for the treatment of liver diseases.

## Background

The liver is involved in regulation of several major physiological processes, such as glycogen storage, lipid metabolism, plasma protein secretion, and xenobiotic detoxification [1]. Liver dysfunction and failure can have diverse etiologies. Orthotropic liver transplantation (OLT) is considered the most suitable therapeutic option for patients with liver failure. However, it is severely limited by organ shortages, high expense, graft rejection, and the requirement for long-term immunosuppression.

Cell-based therapy has been proposed as a potential alternative to OLT [2–4]. Over the past decade, mesenchymal stem cells (MSCs) have attracted considerable attention. MSCs are defined as adherent multipotent fibroblast-type stem cells with the ability to differentiate into mesodermal and ectodermal cells [5, 6]. Unlike other types of stem cells (such as embryonic stem cells and induced pluripotent stem cells), MSCs have low immunogenicity and marked immunomodulatory effects, which reduce the probability of immune rejection [7–9]. Moreover, MSCs are resistant to reactive oxygen species in vitro, reduce oxidative stress in recipient mice, and accelerate repopulation of hepatocytes after liver damage [10]. Therefore, pre-clinical and clinical trials have been performed to determine the therapeutic potential of MSCs [11, 12].

MSCs are distributed extensively and were initially identified in bone marrow [13] and then in various tissues, including the lung, umbilical cord, and adipose tissue [14, 15]. The liver is a novel reservoir of MSCs. Liver-derived human MSCs (LHMSCs) possess properties similar to those of MSCs from other tissues, including proliferative, differentiation, and immunomodulatory capacities. However, LHMSCs are different in certain respects, particularly in terms of their biomarkers and biological functions. This review focuses on hepatic differentiation of LHMSCs and their application in liver disorders, opening a new path toward further studies.

## Isolation and culture of LHMSCs

LHMSCs were first isolated from first-trimester fetal livers [16] and later from second-trimester fetal livers [17]. To ensure the safety, quality, and identity of cell products, a standardized procedure in compliance with current Good Manufacturing Practices has been formulated [18]. Briefly, disrupted liver tissue is harvested using a homogenizer following removal of adjacent tissues. Then, mononuclear cells are isolated by density-gradient centrifugation and cultured in Dulbecco’s modified Eagle’s medium with 15 % fetal bovine serum.

The fetal origin of MSCs raises both ethical and safety issues. Thus, there was much enthusiasm over the isolation of MSCs from adult tissues, which develop and maintain their own stem cell pools. Evidence for the presence of MSCs in the adult liver has accumulated. Najimi et al. [19] successfully obtained adult liver-derived human MSCs by enzymatic disaggregation of adult human liver and the elimination of hepatocytes and other liver cell types. Moreover, Pan et al. [20] reported that these cells are likely a resident population rather than bone marrow-derived cells.

## Characterization of LHMSCs

**Fig. 1:**
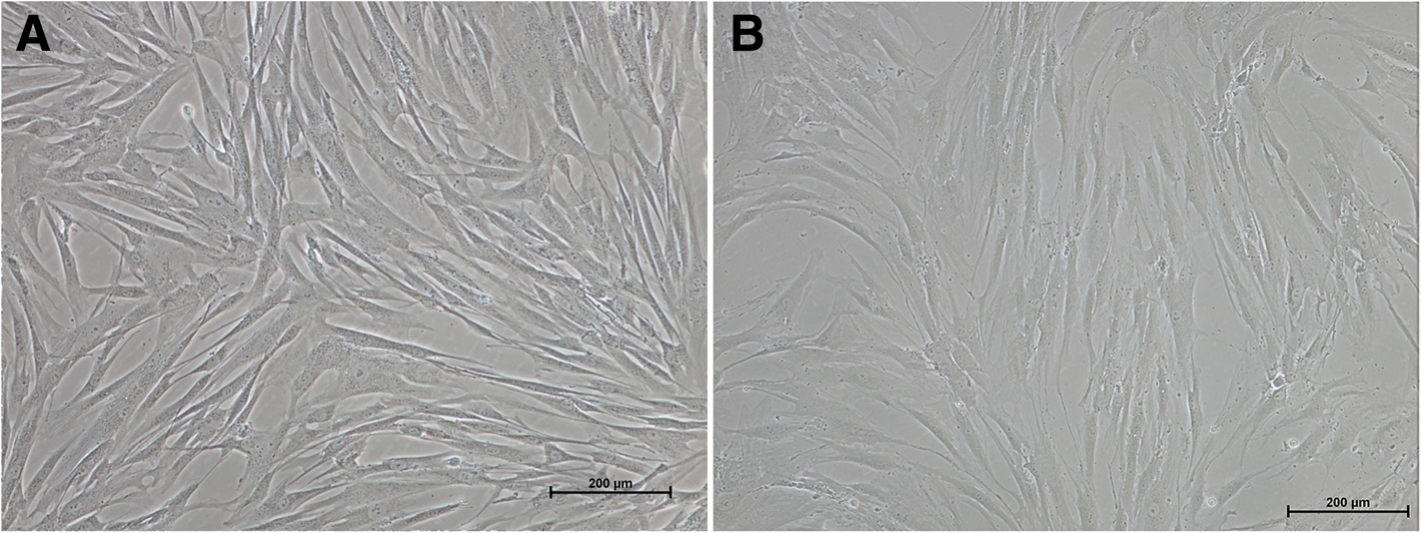
Comparison of the morphology of LHMSCs (**a**) and BMMSCs (**b**). Similar to BMMSCs, LHMSCs are spindle-shaped with ovoid nuclei. Both cells are at passage 3. Original magnification: 100×

In terms of morphology, cultured LHMSCs exhibit an elongated spindle shape with ovoid nuclei (Fig. 1), as well as stem cell properties, including positivity for stem cell markers (vimentin and nestin) and MSC markers (CD29, CD73, CD44, CD90, CD105, and CD166) [21]. However, LHMSCs are negative for hematopoietic stem cell markers (CD34, CD45, CD117), suggesting that these cells are not of hematopoietic origin [19, 22]. Compared with bone marrow-derived MSCs (BMMSCs), the expression of CD105, a marker used to evaluate the differentiation status of MSCs [23], is lower in LHMSCs. This observation suggests that LHMSCs may be at a more advanced stage of differentiation. Interestingly, LHMSCs express CD26, albumin, CK8, and CK18, indicating a partial commitment toward hepatic cell differentiation [21, 22, 24]. Similar to other MSCs, LHMSCs have low immunogenicity due to the absence of major histocompatibility complex (MHC) class II (human leukocyte antigen (HLA)-DP, -DQ, and -DR) antigens, FAS ligand or costimulatory molecules with the exception of MHC class I antigens [25, 26].

Similar to MSCs from other tissues, LHMSCs have the capacity for self-renewal, multipotent differentiation, and immunosuppression. LHMSCs exhibit high proliferative ability in long-term culture, and Wnt signaling has been shown to modulate their growth [20]. In conditioned media, LHMSCs are able to undergo osteogenic, chondrogenic, and endothelial but not adipogenic differentiation. Moreover, LHMSCs can differentiate into hepatocyte-like cells, gaining hepatic functions such as production of cytochrome P450, albumin, and urea. This implies the potential of LHMSCs in cell therapy and pharmacotoxicological testing [22, 27]. It is noteworthy that the proliferative and differentiation capacities of MSCs decrease with age [28, 29]. Thus, fetal LHMSCs may be superior. With regard to their immune effects, LHMSCs express HLA-G [30] and CD90 [22, 29], which regulate immune responses by inhibiting T-cell proliferation [31]. Furthermore, CD90 is stably expressed in LHMSCs, suggesting that such cells exert immunosuppressive effects [32, 33].

LHMSCs differ from other resident hepatic stem/progenitor cells. Dormant liver progenitor cells are periportally located in the healthy liver and actively proliferate after chronic liver injury or sub-massive liver cell loss [34]. LHMSCs are spindle-shaped, whereas liver progenitor cells are oval. Moreover, LHMSCs are negative for CD117, CD34, and CK19, markers of liver progenitor cells [21]. Hepatic stellate cells (HSCs) are another type of stem/progenitor cell in the liver that acquire a myofibroblast-like phenotype when activated. As is true of LHMSCs, activated HSCs express CD133, a molecular maker of stem/progenitor cells [35]. However, HSCs are positive for NCAM, CK19 and HLA-class II membrane markers [36], for which LHMSCs are negative. Moreover, chemokine levels differ markedly between LHMSCs and HSCs: the former cells secrete higher levels of therapeutic and immune-modulatory cytokines, including hepatocyte growth factor (HGF), interferon (IFN)-γ and interleukin (IL)-10 [37].

## Hepatic differentiation protocol

**Fig. 2:**
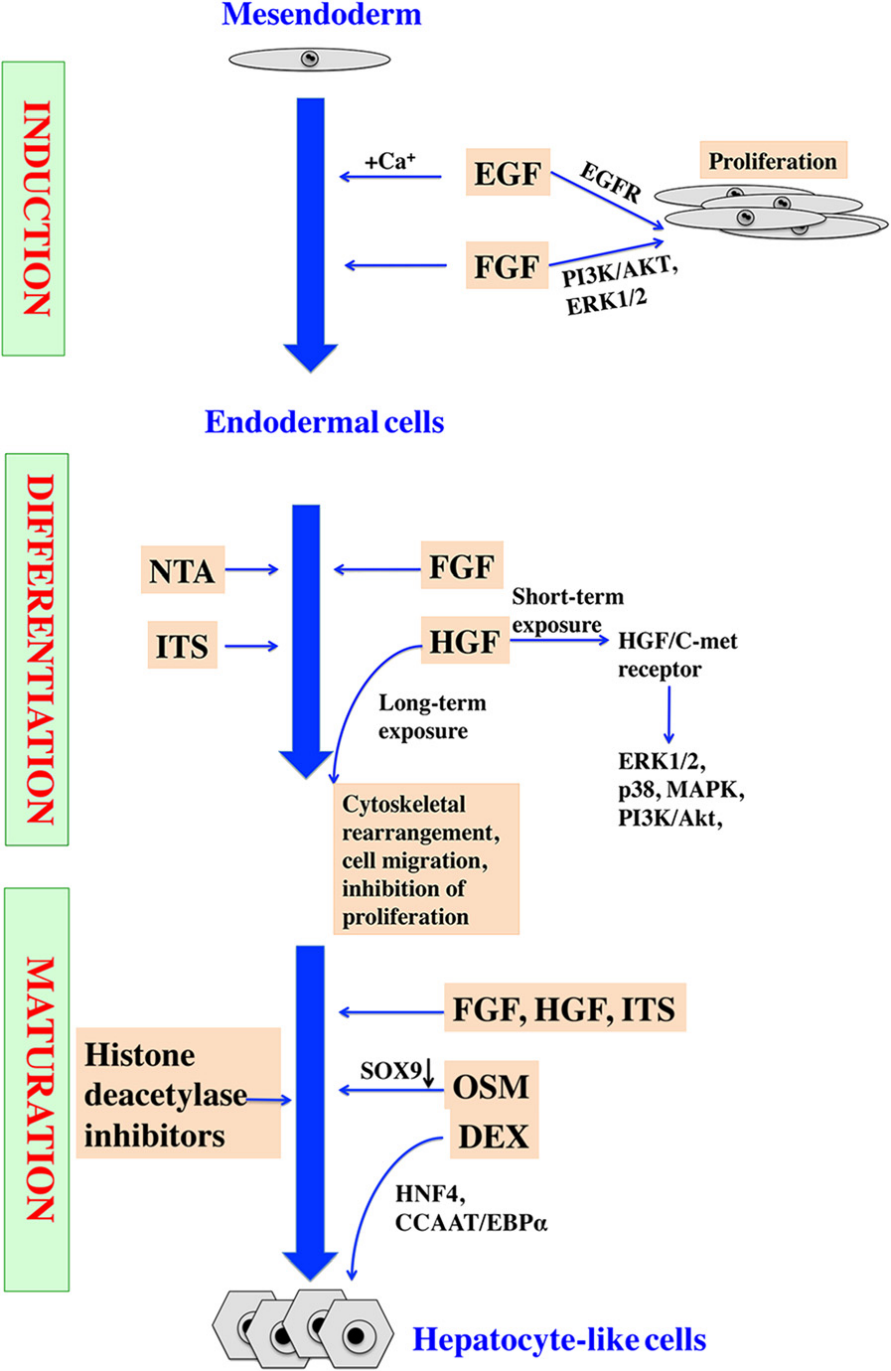
Signaling pathways of LHMSC differentiation into hepatocytes in vitro. Hepatic differentiation can be divided into three stages: induction, differentiation and maturation. Sequential addition of cytokines to the medium plays a vital role at each stage. *DEX* dexamethasone, *EBP* enhancer binding protein, *EGF* epidermal growth factor, *EGFR* epidermal growth factor receptor, *ERK1/2* extracellular-signal-regulated kinase 1/2, *FGF* fibroblast growth factor, *HGF* hepatocyte growth factor, *HNF4* hepatocyte nuclear factor 4, *ITS* insulin-transferrin-selenium, *MAPK* mitogen-activated protein kinase, *OSM* oncostatin M, *PI3K* phosphoinositide 3-kinase, *NTA* nicotinamide

Iscove’s modified Dulbecco’s medium with sequential cytokine supplements (Fig. 2) is the most frequently used hepatic differentiation procedure.

In the initial induction step, MSCs are induced into endodermal cells by epidermal growth factor (EGF) and fibroblast growth factor (FGF). EGF stimulates proliferation of MSCs by binding to EGF receptor (EGFR) [38]. MSCs transfected with an EGF vector and stimulated by Ca^+^ can differentiate into epithelial-like cells [39]. FGF, which constitutes a family of at least seven closely related polypeptides with heparin-binding properties, plays a pivotal role during the initial stage of endodermal patterning [40]. Among these, FGF-4 and basic FGF are used conventionally. Similar to EGF, FGF also increases the proliferation rate of MSCs [41–43].

FGF, HGF, nicotinamide (NTA) and insulin-transferrin-selenium (ITS) are commonly added to cultures to trigger cell differentiation. HGF is a pleiotropic cytokine of mesenchymal origin involved in the regulation of proliferation, differentiation, and chemotactic migration of MSCs [44, 45]. Ghaedi et al. [46] cultured adipose stem cells on HGF/collagen I spots for 2 weeks and found increased expression of hepatocyte-specific genes, indicating hepatic induction of HGF. Interestingly, Forte et al. [45] showed that short-term exposure of MSCs to HGF results in activation of the c-met receptor and the downstream effectors, ERK1/2, p38, MAPK, and PI3K/Akt, while long-term exposure resulted in cytoskeletal rearrangement, cell migration, and marked inhibition of proliferation. ITS and NTA promote the proliferation and survival of primary hepatocytes [47, 48]. Chivu et al. [49] compared the differentiation efficacy of various cytokines, including HGF, ITS, dexamethasone and NTA, and reported HGF and NTA to be the most potent inducers.

To induce further maturation, oncostatin M (OSM) and dexamethasone are required, together with the addition of FGF, ITS, and HGF. Zhou et al. [50] demonstrated that HGF promoted a mid/late hepatic phenotype but failed to induce functional hepatocyte maturation. Thus, a further maturation procedure is needed. OSM is a member of the IL-6 subfamily that plays an important role in progression from hepatocyte development to liver maturation [51, 52]. A recent study indicated that the hepatic induction effects of OSM might be correlated with downregulation of sox 9 [53], which enforces proliferation and maintains the pluripotency of stem/progenitor cells [54, 55]. Dexamethasone induces the expression of both HNF4 and CCAAT/EBPα. Both transcription factors are essential for hepatocyte differentiation [56]. Histone deacetylase inhibitors, such as trichostatin A and sodium butyrate, contribute to hepatic differentiation of stem cells [57–60]. Notably, histone deacetylase inhibitors enhance the expression of hepatocyte-specific genes and functions [61] but decrease the adipogenic, chondrogenic, and neurogenic differentiation potential of MSCs [62].

Although several hundred studies have demonstrated the generation of hepatocyte-like cells, the procedure for identifying a differentiated cell as a hepatocyte has not been standardized. The following sequence of tests is recommended to determine the generation of hepatocytes: 1) quantitative reverse transcription polymerase chain reaction (PCR); 2) protein expression evaluation; 3) ultrastructural evaluation; 4) functional analysis; and 5) engraftment, differentiation, and functional repopulation in vivo [63].

## Cell therapy for liver disorders

MSCs are considered ideal candidates for cell transplantation due to their immunosuppressive, angiogenic, and anti-inflammatory activities. We discuss the use of LHMSCs and LHMSC conditioned medium (LHMSC-CM) in animal models (Table 1) and the clinic.

**Table 1:** Preclinical studies using HLMSCs or HLMSC-CM to treat liver diseases

Cell type	Number of cells infused	Model	Animal	Administration route	Follow-up period	Efficacy	Ref.
LHMSCs	2.5 × 10^6^	Rigler-Najjar type I syndrome	Gunn rats	iv (portal vein)	6 months	Decrease in bilirubin level	[64]
LHMSCs	1 × 10^6^	20 % hepatectomy	SCID mice	ip	60 days	Proliferation and differentiation of LHMSCs in vivo	[66]
LHMSCs	1 × 10^6^	70 % partial hepatectomy	uPA+/+-SCID, SCID mice	ip	56 days	Proliferation and differentiation of LHMSCs in vivo	[19]
LHMSC-CM	–	70 % partial hepatectomy	C57BL/6 mice	Not mentioned	2 days	Enhanced liver regenerative responses	[73]
LHMSCs	5 × 10^5^	Liver fibrosis	NOD/SCID/IL-2Rγ (null) mice	iv (tail vein)	8 weeks	No benefits observed	[87]
LHMSCs	2 × 10^5^	Acute liver injury	SCID mice	iv	30 days	Proliferation of LHMSCs in vivo	[22]
LHMSCs from liver graft preservation fluids	1× 10^6^	Acute liver injury	NOD/SCID mice	ip	4 weeks	Differentiation of LHMSCs in vivo	[20]
MSCs, LHMSC-CM	2 × 10^6^ (iv), 3 × 10^7^ (ip), 5 × 10^5^ or 2 × 10^5^ (LP)	Acute liver failure	SCID mice	iv, ip, LP	21 days	Increased survival rates, decrease in liver metabolic enzymes and ammonium	[81]

*ip* intrasplenic injection, *iv* intravenous injection, *LHMSC* liver-derived human mesenchymal stem cell, *LHMSC-CM* liver-derived human mesenchymal stem cell conditioned medium, *LP* injection via liver parenchyma, *MSC* mesenchymal stem cell

### Metabolic disorders

The liver has a capacity for biotransformation. Khuu et al. [27] evaluated the abilities of differentiated LHMSCs to synthesize glucose and to metabolize ammonia as well as xenobiotics. These functions were enhanced after hepatic induction, suggesting the feasibility of using these cells as alternatives to mature hepatocytes for in vitro toxicopharmacological screening. With respect to the activity of phase II drug-metabolizing enzymes, LHMSCs were transplanted into rodents with Crigler-Najjar syndrome to correct hyperbilirubinemic conditions [64]. Furthermore, Baruteau et al. [65] demonstrated high l-phenylalanine hydroxylase (PAH) expression and a marked increase in PAH activity in differentiated LHMSCs, suggesting therapeutic potential for phenylketonuria.

Pre-clinical safety experiments have not shown any increasing risk of tumorigenicity, either in vitro or in vivo [66]. Thus, these cells have been used in clinical trials. Although LHMSCs exhibit immunosuppressive activities, immunosuppressants cannot be ignored. A patient with glycogenosis type 1A was intraportally injected with 3 billion ^111^InDTPA-labeled cells and no signal was observed in organs other than the liver, suggesting the feasibility of the therapeutic use of LHMSCs [67]. The engraftment potential of LHMSCs has been explored in patients with ornithine carbamoyltransferase deficiency [68] (clinicaltrials.gov identifier NCT01765283, NCT02489292, NCT02051049).

### Liver regeneration

**Fig. 3:**
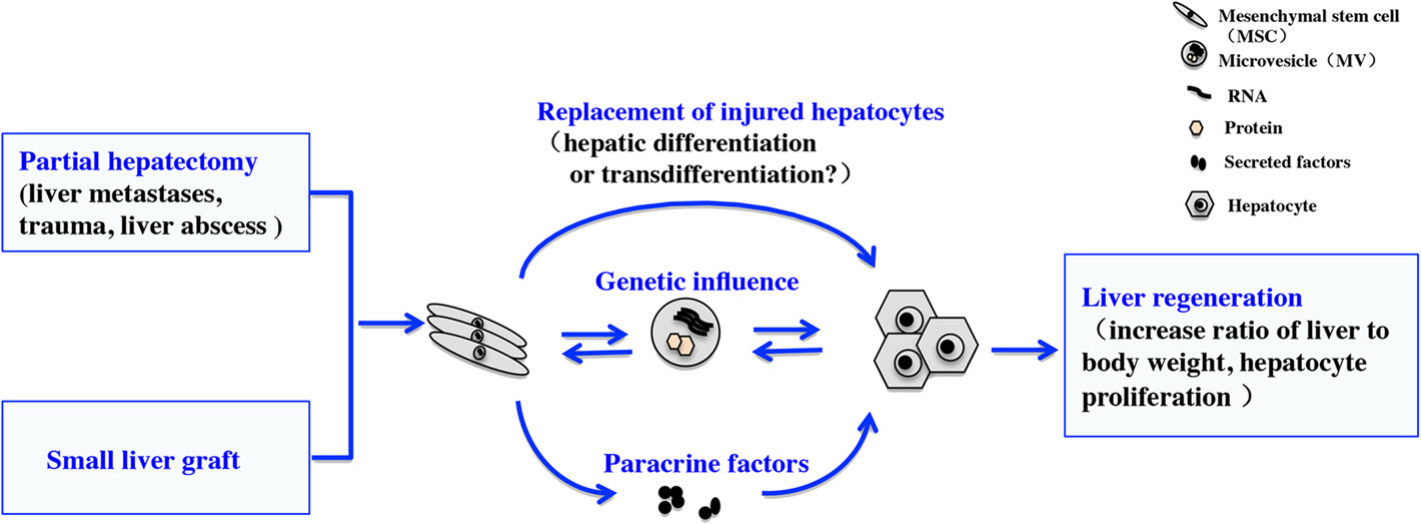
Possible mechanisms of liver regeneration related to LHMSCs. LHMSCs promote regeneration of the remaining liver, likely through the following mechanisms: replacement of injured hepatocytes, secretion of beneficial factors, and genetic modification

The liver is unique in terms of its regeneration ability. Depending on the proliferation of residual mature hepatocytes that re-enter the cell cycle and proliferate, normal liver weight can be re-established within 8–15 days in humans (5–7 days in rodents) [69]. The two-thirds partial hepatectomy rodent model is the classic model used to study liver regeneration. Although LHMSCs have been demonstrated to participate in liver regeneration [19, 70], the mechanisms involved have not been clearly elucidated (Fig. 3).

The effect might be due to hepatic differentiation of multipotent stem cells. In situ hepatic differentiation was observed in transplanted mice, which supports this hypothesis. Nevertheless, other data indicate that cells with donor markers and liver-specific markers in the recipient liver are a product of cell fusion rather than a real transdifferentiation [71, 72].

Paracrine signaling is another possible explanation. MSCs secrete various growth factors, cytokines, and chemokines, of which CCL7, vascular endothelial growth factor (VEGF), and CXC family members are involved in anti-inflammatory responses, apoptosis prevention, and angiogenesis [37]. Because secreted factors have no risk of rejection or malignant transformation, LHMSC-CM might be more beneficial than MSCs. In pre-clinical experiments, the efficacy of application of LHMSC-secreted factors in 70 % hepatectomized mice suggests their potential use in patients undergoing extensive liver resection or transplantation of small liver grafts [73].

The genetic influence of microvesicles (MVs) has also been investigated. MVs are heterogeneous circular membrane fragments that comprise two major populations: exosomes and microparticles. Exosomes originate from the exosomal compartment and are 30–100 nm in diameter. In contrast, microparticles are released directly from budding of the plasma membrane surface and are 100 nm to 1 μm in diameter. The biological significance of MVs was largely overlooked for many years. Recently, they have been recognized to carry proteins, microRNAs, and mRNAs, and to play important roles in cell-to-cell communication [74]. Quesenberry et al. [75] proposed a novel concept known as areas of influence, which refers to the influence of complex effectors on the lability of the stem cell phenotype. Areas of influence include cell cycle passage, complex interactions with stromal cells, and MV-mediated cell-to-cell transfer of genetic information. Stem cells undergo functional and phenotypic changes after receiving genetic information, transferred by MVs, from injured cells. Moreover, feedback from stem cells alters the functions of target cells, suggesting that stem cells repair damaged tissues without directly replacing parenchymal cells [76, 77]. The protective effect of MVs from LHMSCs was confirmed by Herrera et al., who reported that MVs shuttled mRNAs into hepatocytes of hepatectomized rats and accelerated hepatic regeneration [78].

### Acute liver injury

Acute liver failure, a lethal clinical syndrome, is characterized by rapid development of hepatocellular dysfunction with diffuse intrahepatic infiltration of inflammatory cells and massive multilobular necrosis. Based on their release of trophic and immunomodulatory factors, MSCs are commonly used in the treatment of acute liver injury. Parekkadan et al. [79, 80] postulated that soluble factors present in MSC-conditioned medium (IL-6, VEGF, and HGF) were responsible for both local and systemic therapeutic effects. Herrera et al. [81] reported that LHMSCs also significantly prevented death in a fatal model of fulminant liver failure. They further stated that the therapeutic effect was due to a paracrine mechanism.

### Liver cirrhosis

Liver cirrhosis, the most advanced stage of fibrosis, connotes not only more scarring than that from fibrosis alone, but also distortion of the liver parenchyma associated with septae and nodule formation, altered blood flow, and risk of liver failure [82]. The activation of HSCs is a pivotal event in the development of liver cirrhosis [12]. It seems that the anti-fibrotic effects of MSCs in liver cirrhosis are based on the release of factors that alter the function of HSCs. HGF is expressed highly in LHMSCs. Overexpression of HGF promotes HSC apoptosis [83, 84]. Moreover, HGF is associated with hepatogenesis. The plasma HGF level increases considerably after partial hepatectomy [85]. Compared with BMMSC conditioned medium, the HGF level was ~50-fold higher in LHMSC-CM [81]. IL-10 and tumor necrosis factor (TNF)-α also reduce the proliferation of HSCs and synthesis of collagen type I [37, 79]. Moreover, LHMSCs can secrete IFN-γ, inducing anti-fibrotic effects [86].

However, in a murine model of CCl4-induced liver fibrosis, intravenous administration of LHMSCs failed to improve liver function [87] and the injected cells propagated in various tissues; less effective transplantation may explain the failure. To promote MSCs homing to the liver, the following approaches can be employed: 1) direct injection, such as injection via the portal vein, spleen, and liver parenchyma; and 2) use of MSCs modified by liver-specific receptors.

## Conclusions

As a novel type of MSC, LHMSCs exhibit general properties of MSCs, including self-renewal, multipotent differentiation and immunomodulation. Compared with other types of MSCs, LHMSCs have several advantages, such as more secretion of protective factors. However, the following problems must be addressed: 1) how to induce differentiation of LHMSCs into functional cells that are more similar to primary hepatocytes; 2) identifying the criteria for evaluation of the degree of hepatic differentiation of LHMSC-derived cells; 3) improvement of the efficiency of cell homing to the liver after transplantation; 4) determining the number of cells required for various diseases; and 5) identifying the possible risks of LHMSC transplantation. Thus, further studies are needed to characterize LHMSCs, improve the efficacy of hepatic differentiation, and validate their therapeutic potential in liver diseases.

## Abbreviations

BMMSC: bone marrow-derived mesenchymal stem cell
EGF: epidermal growth factor
EGFR: epidermal growth factor receptor
FGF: fibroblast growth factor
HGF: hepatocyte growth factor
HLA: human leukocyte antigen
HSC: hepatic stellate cell
IFN: interferon
IL: interleukin
ITS: insulin-transferrin-selenium
LHMSC-CM: liver-derived human mesenchymal stem cell conditioned medium
LHMSC: liver-derived human mesenchymal stem cell
MHC: major histocompatibility complex
MSC: mesenchymal stem cell
MV: microvesicle
NTA: nicotinamide
OLT: orthotropic liver transplantation
OSM: oncostatin M
PAH: l-phenylalanine hydroxylase
VEGF: vascular endothelial growth factor
